# E-Point Septal Separation Accuracy for the Diagnosis of Mild and Severe Reduced Ejection Fraction in Emergency Department Patients

**DOI:** 10.24908/pocus.v7i1.15220

**Published:** 2022-04-21

**Authors:** José Atilio Núñez-Ramos, María Camila Pana-Toloza, Sheyla Carolina Palacio-Held

**Affiliations:** 1 Emergency Department, Hospital Universidad del Norte; Health Division Sciences Universidad del Norte Barranquilla Colombia

**Keywords:** E-Point Septal Separation, Accuracy, Left Ventricle Ejection Fraction, Emergency Room

## Abstract

**Introduction**: In the Emergency Department (ED), a thorough cardiovascular evaluation cannot be accomplished only with physical examination. E-Point Septal Separation (EPSS) measure through Point-of-Care Ultrasound (POCUS) has been used to evaluate systolic function in echocardiography. We analyzed EPSS for diagnosis of Left Ventricle Ejection Fraction <50% and ≤40% in ED patients. **Methods**: Retrospective analysis of a convenience sample of patients presenting to ED with chest pain or dyspnea who underwent admission POCUS evaluation by Internal Medicine Specialist unaware of Transthoracic Echocardiogram. Accuracy was assessed with sensitivity, specificity, likelihood ratios (LR) and Receiver operating characteristics (ROC) curve. The best cut off point was calculated using Youden Index. **Results**: Ninety-six patients were included. Median EPSS and LVEF were 10mm and 41% respectively. Area Under the ROC Curve (AUC-ROC) to diagnose a LVEF <50% was 0.90 (IC95% 0.84-0.97). Youden Index was 0.71 with cut off point EPSS at 9.5mm, performing with a sensitivity of 0.80, a specificity of 0.91, a positive LR of 9.8 and a negative LR of 0.2. AUC-ROC to diagnose a LVEF ≤40% was 0.91 (IC95% 0.85-0.97). Youden Index was 0.71 with a cut off point EPSS at 9.5mm, performing with a sensitivity of 0.91 and specificity of 0.80, a positive LR of 4.7 and a negative LR of 0.1. **Conclusion**: EPSS can reliably diagnose reduced LVEF in a set of ED patients with cardiovascular symptoms. A cut off point at 9.5 mm has good sensitivity, specificity and Likelihood ratios.

## Introduction

Chest pain, dyspnea and syncope are among the most common reasons to seek care in the Emergency Department (ED). Chest pain accounts for more than a thousand visits per year 1, dyspnea and syncope represent approximately 7 to 8% of ED consults 2, 3. At this moment, a thorough cardiovascular evaluation cannot be accomplished only with physical examination. Valvular disease and systolic dysfunction diagnosis improve when evaluated with a physical exam along with cardiac ultrasound 4.

For the past decade, Point-Of-Care-Ultrasound (POCUS) has become a widely available tool to evaluate ED patients and discriminate high-risk diagnosis and initiate appropriate treatment 5. One of the most sensitive evaluations is cardiac function, specifically the assessment of systolic function. For a complete cardiovascular evaluation, it is important to establish Left Ventricular Ejection Fraction (LVEF), given that it represents prognosis, medical treatment and eventually invasive interventions according to clinical presentation. LVEF of less than 40% represent reduced systolic dysfunction, thus poorer outcomes and worse prognosis, with the need of specific medications such as mineralocorticoid receptor antagonists (MRA), Angiotensin Receptor Neprilysin Receptor Inhibitor (ARNI) among others 6. Recently, LVEF of less than 50% has been defined as a mildly reduced ejection fraction, becoming an issue to evaluate in cardiovascular patients in the Emergency Department 7. 

E-Point Septal Separation (EPSS) measure has been used to evaluate systolic function in echocardiography 8. The strong correlation of EPSS and LVEF has been assessed with adequate results. Its limitations are known and include mitral stenosis, hypertrophic cardiomyopathy, and aortic regurgitation. There are studies evaluating the accuracy of EPSS to predict a LVEF <30% in dyspneic patients and <50% in perioperative elective patients 9, 10, but there is scarce information about accuracy of EPSS for the diagnosis of LVEF less than 50% and 40% in ED patients consulting for cardiovascular symptoms.

We analyzed ED patients presenting with chest pain and dyspnea who received a formal transthoracic echocardiogram (TTE) and POCUS at admission. This study aimed to evaluate the diagnostic accuracy of EPSS to predict reduced (LVEF ≤40%) and mildly reduced Ejection Fraction (LVEF < 50%).

## Methods

This is a retrospective study including Emergency Room patients older than 18 years with cardiovascular symptoms in a Tertiary University-based Hospital which receives approximately 70.000 emergency visits per year. The Ethics Committee and Institutional Board approved this protocol (Act 244 July 2021).

Patients with chest pain or dyspnea as chief complaint and consulting our Emergency Room from July 2019 to March 2021 were identified through chart review. Those who received POCUS evaluation at admission and a transthoracic echocardiogram during the hospitalization were eligible. Patients with shock or hypotensive at admission were excluded, as well as patients in cardiac arrest at admission or those who received inotropes and/or vasopressors. 

**Figure 1:**
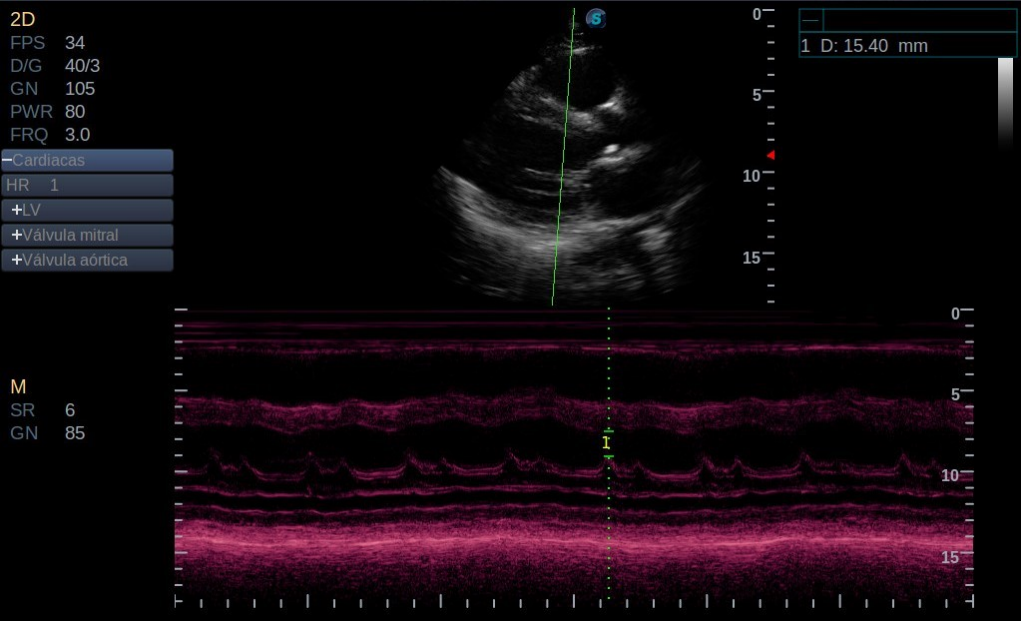
E-Point Septal Separation Measure. The E-Point Septal Separation measure was taken in early diastole, using Parasternal Long Axis View (PLAX) M-Mode, between the tip of the anterior mitral valve leaflet and the interventricular septum.

POCUS evaluation was performed at admission by the attending Internist as part of usual care. The equipment used was a Sonoscape S2 Ultrasound Machine with a 2.5MHz Phased Array (Sonoscape Corp. Guangdong, China. 2016-3). The E-Point Septal Separation measure was taken in early diastole, using Parasternal Long Axis View (PLAX) M-Mode, between the tip of the anterior mitral valve leaflet and the interventricular septum (Figure 1). 

Only one ED Internist with formal POCUS training and 3 years of experience performed ultrasound evaluations. The Internal Medicine (IM) specialist was the treating clinician of this group of patients during their stay in the ED. At admission, IM clinician was aware of chief complaint (chest pain, dyspnea) and the clinical background of patients (Heart Failure, Hypertension, Acute Coronary Syndrome) but was unaware of previous LVEF. 

Transthoracic echocardiogram was performed by a cardiologist with a subspecialty in echocardiography and more than 10 years of experience. LVEF was calculated through Simpson Biplane formula. Given the patient flow in our hospital, IM Clinician performed POCUS evaluation before TTE, therefore Cardiologist and IM POCUS performing were unaware of each other evaluation. 

Main outcome was diagnostic accuracy of EPSS for LVEF in a formal echocardiogram performed by an experienced Cardiologist. We evaluated sensitivity, specificity, likelihood ratios and we performed a receiver operating characteristics curve to establish the best cut off point for a LVEF ≤40% and <50%.

### Statistical analysis

All clinical characteristics were collected from electronic records. Qualitative variables were analyzed using frequencies and percentages. Quantitative variables were reported as median with interquartile ranges due to non-parametric distribution. EPSS measurements were reported as a continuous variable in millimeters. Diagnostic accuracy was evaluated with sensitivity, specificity, likelihood ratios and Youden Index (YI). Receiver operating characteristics (ROC) curves were obtained for the prediction of an Ejection Fraction (EF) ≤40% and <50%. The best cut off point was calculated through the YI. All analyses were performed using SPSS Software Version 25. 

## Results

A total of 96 patients were included in the analysis. Basal characteristics are shown in Table 1. Median age was 61 years, most patients (61.5%) were male. The most common comorbid condition was hypertension (60.4%) followed by type 2 diabetes mellitus (24%). The main chief complaint at admission was dyspnea and chest pain (96%). A total of 24 (25%) patients had Acute Coronary Syndrome as admission diagnosis and 62.5% were diagnosed with acute decompensated heart failure. The 51.2% of individuals had myocardial injury (positive troponin I test). Among all patients included, 46.9% had a normal ECG, 5.2% ST-segment elevation, 12.5% ST-segment depression, 5.2% atrial fibrillation and 14.6% any bundle-branch block. 

**Table 1:** Basal Characteristics.

	n=96
**Demographics and Medical History **	
Age	61 (52-76)
Male	59 (61,5)
Hypertension	58 (60,4)
Type 2 Diabetes	23 (24)
Heart Failure	16 (16,7)
Acute Coronary Syndrome	12 (12,5)
**Admission Diagnosis **	
Acute Coronary Syndrome	24 (25)
Acute Decompensated Heart Failure	60 (62,5)
COVID-19 Pneumonia	9 (9,5)
Others (Pleural Effusion and Pulmonary Embolism)	3 (3)
**Clinical Variables **	
Systolic Blood Pressure	140 (119-167)
Median Blood Pressure	99 (88-122)
Heart Rate	94 (78-110)
Pulse Oximetry	97 (92-98)
Creatinine	1,2 (1-2)
B-Type Natriuretic Peptide	1076 (98-2550)
Positive Troponin I	43 (44.8)
Normal EKG	45 (46.9)
ST Segment Elevation / Depression	17 (17.7)
*Categorical variables are expressed in absolute frequency and (%) percentages. Quantitative variables are expressed in median (interquartile ranges).	

The median for EPSS and LVEF was 10 mm and 41% respectively. The prevalence of reduced systolic function (LVEF ≤40%) was 45.8% and prevalence for mildly reduced ejection fraction (LVEF <50%) was 61.5%. The median time between POCUS evaluation and TTE was 5.5 hours (Interquartile range 2 - 24 hours). Table 2 presents ultrasound findings for POCUS and TTE. 

**Table 2:** POCUS and Transthoracic Echocardiogram Findings.

	n=96
**POCUS **	
EPSS measure (mm)	10 (6-17)
Qualitative Depressed Systolic Function	43 (44,8)
Dilated Right Cavities	9 (9,4)
Pericardial Effusion	15 (15,6)
B lines	72 (75)
**Transthoracic Echocardiogram **	
LVEF (%)	41 (27-56)
EF <50%	59 (61,5)
EF ≤40%	44 (45,8)
Time to TTE	5,5 (2-24)
*Categorical variables are expressed in absolute frequency and (%) percentages. Quantitative variables are expressed in median (interquartile ranges).	

**Figure 2:**
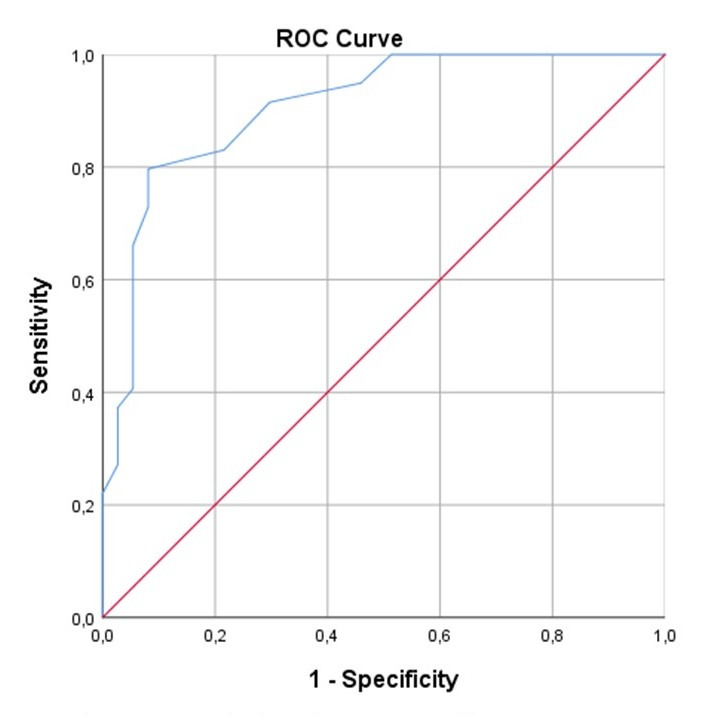
ROC Curve of EPSS for diagnosis of LVEF <50%. ROC Curve showing the Area Under the Curve calculated 0.90 (IC95% 0.84-0.97) for EPSS to diagnose LVEF <50%.

The Area Under the Curve (AUC) of the ROC Curve (AUC-ROC) for EPSS to diagnose a LVEF <50% was 0.90 (IC95% 0.84-0.97). The highest Youden Index was 0.71 with a cut off point EPSS at 9.5 mm, performing with a sensitivity of 0.80, a specificity of 0.91, a positive LR of 9.8 and a negative LR of 0.2. (Figure 2)

**Figure 3:**
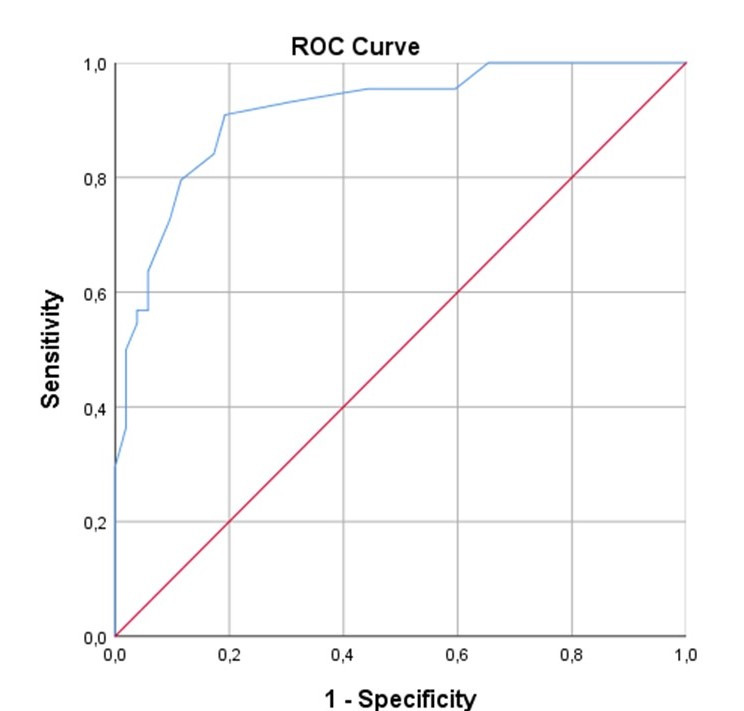
ROC Curve of EPSS for diagnosis of LVEF ≤40%. ROC Curve showing the Area Under the Curve calculated 0.91 (IC95% 0.85-0.97) for EPSS to diagnose LVEF ≤40%.

The Area Under the Curve (AUC) of the ROC Curve (AUC-ROC) for EPSS to diagnose a LVEF ≤40% was 0.91 (IC95% 0.85-0.97). The highest Youden Index was 0.71 with a cut off point EPSS at 9.5mm, performing with a sensitivity of 0.91 and specificity of 0.80, a positive LR of 4.7 and a negative LR of 0.1 (Figure 3).

In Table 3 and 4 are displayed the different EPSS measures with each sensitivity, specificity, Likelihood Ratios and Youden Index. 

**Table 3:** Diagnostic Accuracy of EPSS for LVEF<40%.

**Cut off Point**	**Sensitivity**	**Specificity**	**Youden** **Index**	**Positive** **Likelihood Ratio**	**Negative** **Likelihood Ratio**
4.5	1.000	0.346	0.346	1.529	0.000
5.5	0.955	0.404	0.358	1.601	0.113
6.5	0.955	0.481	0.435	1.838	0.095
7.5	0.955	0.558	0.512	2.158	0.082
8.5	0.932	0.692	0.624	3.028	0.098
**9.5**	**0.909**	**0.808**	**0.717**	**4.727**	**0.113**
10.5	0.841	0.827	0.668	4.859	0.192
11.5	0.795	0.885	0.680	6.894	0.231
12.5	0.727	0.904	0.631	7.564	0.302
13.5	0.636	0.942	0.579	11.030	0.386

**Table 4:** Diagnostic Accuracy of EPSS for ≤50%.

**Cut off Point**	**Sensitivity**	**Specificity**	**Youden** **Index**	**Positive** **Likelihood Ratio**	**Negative** **Likelihood Ratio**
4.5	1.000	0.486	0.486	1.947	0.000
5.5	0.949	0.541	0.490	2.066	0.094
6.5	0.932	0.622	0.554	2.464	0.109
7.5	0.915	0.703	0.618	3.079	0.121
8.5	0.831	0.784	0.614	3.841	0.216
**9.5**	**0.797**	**0.919**	**0.716**	**9.825**	**0.221**
10.5	0.729	0.919	0.648	8.989	0.295
11.5	0.661	0.946	0.607	12.229	0.358
12.5	0.593	0.946	0.539	10.975	0.430
13.5	0.492	0.946	0.437	9.093	0.538

## Discussion

In this retrospective diagnostic study, we found a good accuracy of E-Point Septal Separation for the diagnosis of mildly reduced and reduced ejection fraction with Echocardiography in patients with cardiovascular symptoms in the Emergency Department. 

A cut off point of 9.5 mm allowed sensitivities of 80% and 91% for mildly reduced EF and reduced EF respectively, along with specificities of 91% and 80% for mildly reduced and reduced EF. Positive likelihood Ratios were 9.8 for mildly reduced EF and 4.7 for reduced EF. 

There are several studies analyzing the accuracy of EPSS for left ventricle dysfunction according to LVEF. McKaigney et al^10^ published in 2014 a prospective study with unselected ambulatory patients with any indication for TTE. This study used a LVEF classification according that moment, and Teichholz Method for LVEF calculation. The cut-off point used was 7 mm with a sensitivity of 100% and specificity of 51% for ≤ 30% LVEF. Our study chose emergency room patients with specific cardiovascular complaints, thus selecting a punctual population very common in emergency rooms. Additionally, we used the most recent LVEF classification and Simpson’s Biplane formula for calculating LVEF, which is the recommended method in guidelines 11. Another study in 2021 evaluated elective preoperative patients 9. A low prevalence (22%) of LVEF <50% was found. EPSS cut off point was 6mm with an AUC-ROC 0.89, sensitivity 86% and specificity 88%. This population is in many ways different from ours, including the low prevalence of left ventricle dysfunction and the use of a different cut off point for the analysis. There is a registered systematic review in PROSPERO database, which is an ongoing review for the level of agreement between emergency physicians and expert echocardiographers, consequently there are no results yet and it is not aimed to evaluate diagnostic accuracy as it was in our study 12. 

We found a similar proportion of patients having depressed left ventricle function in POCUS evaluation (44.8%) compared to TTE of LVEF <40% (45.8%) which could represent the consistency in ultrasound findings between POCUS and TTE formal evaluation. Youden Index and AUC-ROC for LVEF <50% and ≤40% were the same, this reflects the fact that differentiating these two types of patients could be challenging. The EF 40-50% “gray-zone” of mildly reduced EF represents patients in an intermediate stage of HF sharing clinical characteristics of reduced and preserved EF patients 7. We consider patients in the ED do not have specific need for identification mildly reduced or normal EF. In contrast, identifying patients with an EF <40% in the context of Acute Coronary Syndrome or new-onset Heart Failure is important to promote the rapid initiation of treatment and interventions. There are other methods to assess LVEF through POCUS as qualitative evaluation, which focuses on myocardial thickening and fractional shortening, based on calculating left ventricle end-diastole and end-systole diameter. These two methods have limitations such as subjective visual-based estimation for the first and complex formula with no direct correlation with LVEF for the second. 

We actively excluded unstable patients requiring vasopressors or inotropes to control confounding on EF calculation after administering inotropes. In this one-center experience, the median time to TTE was 5.5 hours, which could represent the time one attending saves for the diagnosis and treatment initiation. The interquartile range between 2 hours and 24 hours (sometimes even 96 hours) gives an idea of the long time a patient must wait for a formal echocardiographic evaluation.

The insights of AUC-ROC allow us to consider an EPSS of less than 5.5 mm to rule-out an EF <50% and consequently EF <40% (Negative LR 0.09, Sensitivity 95%). On the other hand, an EPSS ≥13.5 confirms EF <40% (Positive LR 11, Specificity 95%) and an EPSS ≥11.5 confirms EF <50% (Positive LR 12, Specificity 95%). 

Our limitations include a small sample size and the fact that only one ED Internist was capable of performing ultrasound evaluations due to lack of staff training; moreover one cardiologist did all transthoracic echocardiograms. This limitation could positively impact results considering there is no inter-observer variability. At the same time, the situation may reflect a lack of POCUS use among ED specialists. There is a need to promote and ensure access to Point-Of-Care Ultrasound training for healthcare professionals. This study was carried out in one center, thus reducing inference of its results. Our population had a higher prevalence of reduced EF than many other publications, so care must be taken in extrapolating results in other institutions.

Our study has several strengths. We analyzed an emergency room set of cardiovascular patients in need to be evaluated thoroughly to confirm or rule-out left ventricle dysfunction. Our patients would have taken a different treatment and diagnosis path if they would have had or not a depressed LVEF. Patients with dyspnea or chest pain and LVEF less than 40% have different prognosis and management, involving a different decision-making process. The blinding of health-care professionals performing ultrasound evaluations allowed an unbiased evaluation of the test and the gold-standard. All patients included in our analysis underwent POCUS and echocardiogram evaluations (test and gold-standard) as is the recommendation for diagnostic accuracy studies. 

These results impulse the use of EPSS in ED patients, allowing ED Physicians and internists to perform POCUS evaluation with more certainty of establishing accurate Left Ventricle Function and making decisions more appropriate in each case. We also provide a table with cut off values and its sensitivities, specificities and likelihood ratios so every clinician could use the best cut off point according to low-probability or high-probability clinical scenario. EPSS measures should become the standard of care in focused cardiac assessment for patients with chest pain and dyspnea in the emergency room. 

## Conclusion

EPSS is a reliable tool to diagnose reduced LVEF in a set of ED patients with cardiovascular symptoms. A cut off point at 9.5mm has reasonable sensitivity and specificity to diagnose reduced LVEF.

## Statement of ethics approval

At a meeting of the Research Ethics Committee in the Health Area of the University del Norte, made on July 29, 2021, and legalized by act No. 244, the consensus of its members approve the research protocol and waved the informed consent considering this observational research.

## Disclosures

None.

## References

[R156751526277429] Ko D T, Dattani N D, Austin P C (2018). Emergency Department Volume and Outcomes for Patients After Chest Pain Assessment. Circ Cardiovasc Qual Outcomes.

[R156751526277432] Kelly A M, Keijzers G, Klim S (2017). An Observational Study of Dyspnea in Emergency Departments: The Asia, Australia, and New Zealand Dyspnea in Emergency Departments Study (AANZDEM). Acad Emerg Med Off J Soc Acad Emerg Med.

[R156751526277433] Brignole M, Moya A, Lange F J De (2018). ESC Guidelines for the diagnosis and management of syncope. Eur Heart J.

[R156751526277431] Marbach J A, Almufleh A, Santo P Di (2019). Comparative Accuracy of Focused Cardiac Ultrasonography and Clinical Examination for Left Ventricular Dysfunction and Valvular Heart Disease: A Systematic Review and Meta-analysis. Ann Intern Med.

[R156751526277428] Ahn J H, Jeon J, Toh H-C (2017). SEARCH 8Es: A novel point of care ultrasound protocol for patients with chest pain, dyspnea or symptomatic hypotension in the emergency department. PLoS One.

[R156751526277426] Mcdonagh T A, Metra M, Adamo M (2021). 2021 ESC Guidelines for the diagnosis and treatment of acute and chronic heart failure. Eur Heart J.

[R156751526277425] Savarese G, Stolfo D, Sinagra G (2021). Heart failure with mid-range or mildly reduced ejection fraction. Nat Rev Cardiol.

[R156751526277427] Secko M A, Lazar J M, Salciccioli L A (2011). Can junior emergency physicians use E-point septal separation to accurately estimate left ventricular function in acutely dyspneic patients?. Acad Emerg Med Off J Soc Acad Emerg Med.

[R156751526277435] Stenberg Y, Wallinder L, Lindberg A (2021). Preoperative Point-of-Care Assessment of Left Ventricular Systolic Dysfunction With Transthoracic Echocardiography. Anesth Analg.

[R156751526277436] Mckaigney C J, Krantz M J, Rocque La (2014). E-point septal separation: a bedside tool for emergency physician assessment of left ventricular ejection fraction. Am J Emerg Med.

[R156751526277430] Mitchell C, Rahko P S, Blauwet L A (2019). Guidelines for Performing a Comprehensive Transthoracic Echocardiographic Examination in Adults: Recommendations from the American Society of Echocardiography. J Am Soc Echocardiogr Off Publ Am Soc Echocardiogr.

[R156751526277434] Albaroudi Mahmoud Bilal, Haddad Omar, Albaroudi (2021). The level of agreement between emergency physicians and the expert echocardiographers, in assessing the left ventricular systolic function in patient attending emergency department. A systematic review and meta-analysis. https://www.crd.york.ac.uk/prospero/display_record.php?RecordID=179209.

